# Supplementary material for: Physical origin of a complicated tactile sensation: ‘shittori feel'
Source: R Soc Open Sci. 2019 Jul 10;6(7):190039. doi: 10.1098/rsos.190039 (PMC6689606; doi:10.1098/rsos.190039)
Supplement: Physical propertiese of materials [file rsos190039supp1.pdf]

Supplementary material

Physical origin of a complicated tactile sensation "*Shittori* feel"

Kana Kikegawa, Rieko Kuhara, Jinhwan Kwon, Maki, Sakamoto, Reiichiro Tsuchiya, Noboru Nagatani, Yoshimune Nonomura

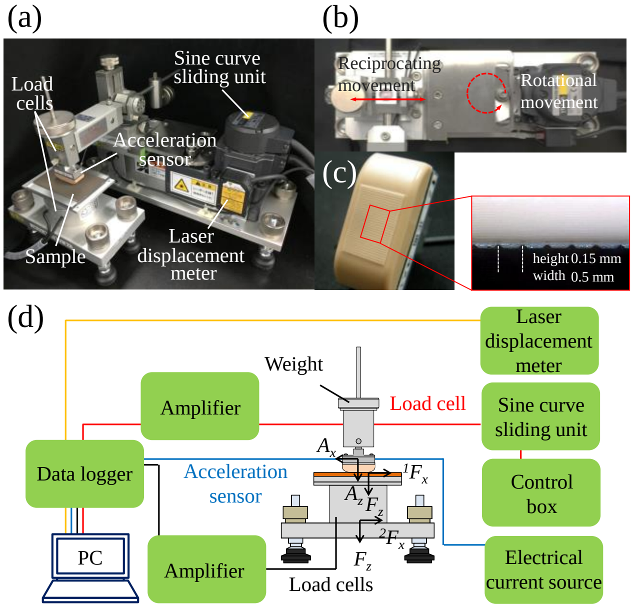

Figure S1 Sinusoidal-movement sliding system. (a) Overall view, (b) scotch yoke mechanism, (c) finger model, (d) conceptual diagram. From ref. 22.

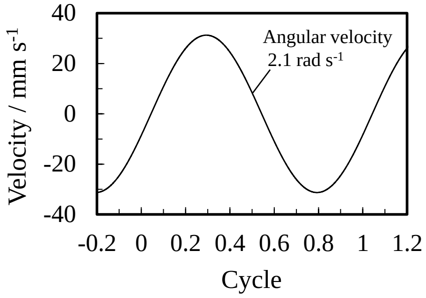

Figure S2 Velocity change during the time of one period for angular velocity 2.1 rad s^{-1}.

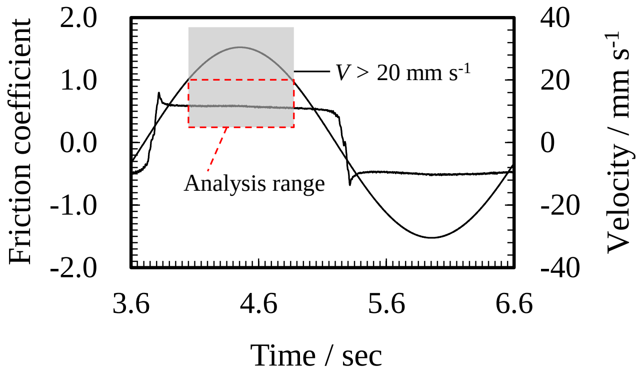

Figure S3 The friction variation value is standard deviation of kinetic friction coefficient. It was analyzed for kinetic friction coefficient in the range of $V > 20$ mm s^{-1}.

Table S1 Sensory scores of 12 materials. Q1: “When you touched the material, did you feel *shittori* feel?” Q3: Evaluations of 10 tactile sensations regarding the tactile dimensions. Timing: If the score of *shittori* feel was 7 or more, the subjects were asked when the *shittori* feel was felt. 1=“The moment when you touch the material,” 2=“while touching,” 3=“the moment when you take your finger off,” and 4=“I do not know.”

A

ID	Q1	Timing				Q3										Mean	Standard deviation
	Shittori	1	2	3	4	Soft	Warm	Rough	Sticky	Moist	Hard	Cold	Smooth	Slippery	Dry		
1	8.1	○	○			7.8	0.0	0.0	10.0	8.1	0.0	1.6	7.1	7.5	7.1	5.2	3.9
2	9.3		○			0.0	1.2	0.0	10.0	7.6	0.1	0.0	8.5	3.9	1.4	3.8	4.2
3	7.4		○			9.2	1.8	0.6	4.5	5.2	0.4	4.0	9.5	1.2	2.3	4.2	3.3
4	7.9		○			6.3	0.9	0.0	6.4	8.4	0.5	0.8	5.4	3.8	2.6	3.9	3.1
5	6.0					4.3	8.2	4.9	4.1	6.7	2.2	0.7	6.2	6.5	1.6	4.7	2.4
6	9.5		○			6.8	0.7	0.7	4.1	7.0	9.1	1.5	9.4	4.5	0.7	4.9	3.6
7	9.9	○	○			7.5	1.7	0.8	8.7	10.0	2.6	6.8	10.0	9.6	2.3	6.4	3.7
8	8.8	○				8.6	4.0	2.2	7.8	8.9	1.4	7.4	2.1	7.7	4.2	5.7	3.0
9	8.7	○	○			8.2	3.1	1.5	8.8	7.8	1.7	2.9	7.0	4.0	2.0	5.1	3.0
10	6.7					8.7	7.4	2.5	6.2	6.6	1.2	3.4	7.4	4.4	3.0	5.2	2.4
11	7.1		○			7.6	4.8	7.4	7.3	7.3	2.6	3.5	5.9	5.0	3.5	5.6	1.8
12	7.8	○	○			8.1	4.4	1.3	7.6	6.2	2.0	4.3	6.3	7.8	3.0	5.3	2.5
13	7.5	○	○			7.3	7.5	0.0	7.9	7.3	3.5	2.9	7.4	7.6	1.9	5.5	2.9
14	8.6		○			7.8	5.8	1.4	2.3	8.7	0.0	2.0	8.8	8.5	0.0	4.9	3.8
15	7.5		○			5.8	5.0	5.7	9.2	8.3	1.8	1.1	3.8	6.7	0.9	5.1	2.9
16	4.8					8.5	0.0	1.8	4.9	3.2	2.2	0.0	6.9	3.6	3.8	3.6	2.6
17	7.3		○			8.6	7.4	1.0	2.8	7.3	0.8	1.0	8.0	7.0	4.2	5.0	3.1
18	7.9		○			9.1	1.8	0.1	0.5	3.9	0.0	0.2	9.6	1.9	0.3	3.2	3.8
19	6.1					9.0	9.4	1.0	5.9	3.2	1.0	1.5	5.9	6.4	2.2	4.7	3.1
20	9.5		○			9.6	1.6	0.2	9.4	8.3	0.3	7.8	9.6	9.5	1.1	6.1	4.2
Mean	7.8					7.4	3.8	1.7	6.4	7.0	1.7	2.7	7.2	5.9	2.4	4.9	3.2
Standard deviation	1.3					2.2	3.0	2.1	2.7	1.9	2.0	2.4	2.1	2.4	1.6	0.8	0.7

B

ID	Q1	Timing				Q3										Mean	Standard deviation
	Shittori	1	2	3	4	Soft	Warm	Rough	Sticky	Moist	Hard	Cold	Smooth	Slippery	Dry		
1	6.7					6.9	3.0	0.0	8.2	0.0	1.6	1.5	10.0	8.5	9.9	5.1	3.9
2	6.9					1.2	0.6	1.0	9.3	3.7	0.5	0.9	7.7	3.5	1.2	3.3	3.2
3	5.7					9.4	5.7	0.8	3.0	1.0	1.1	3.3	8.7	2.3	1.5	3.9	3.1
4	5.0					3.4	0.5	0.9	2.2	3.0	2.0	0.0	10.0	10.0	7.2	4.0	3.6
5	7.8		○			6.2	6.2	0.7	6.9	9.2	0.3	8.0	9.5	9.8	0.6	5.9	3.7
6	7.8		○			1.2	1.1	0.6	2.5	1.3	0.8	7.3	0.8	8.9	3.8	3.3	3.2
7	9.9		○			8.1	7.6	8.9	9.2	10.0	2.6	1.2	9.5	9.9	2.6	7.2	3.4
8	6.4					9.4	3.6	0.6	7.1	7.1	0.1	7.7	2.9	8.0	3.5	5.1	3.1
9	3.3					10.0	5.6	2.6	10.0	6.2	0.9	6.9	6.6	3.5	5.9	5.6	2.9
10	6.7					6.8	4.6	2.1	3.9	6.7	1.9	2.7	7.1	7.6	4.3	4.9	2.1
11	7.2		○			7.7	6.3	1.0	7.9	5.7	1.7	2.2	6.7	7.2	5.1	5.3	2.5
12	7.7	○	○			7.0	1.8	2.0	8.4	3.9	2.2	4.0	7.7	8.6	3.1	5.1	2.8
13	6.0					7.3	6.1	1.2	6.3	3.7	1.9	3.5	8.6	7.6	3.5	5.1	2.4
14	6.2					8.3	6.8	0.1	3.6	7.6	1.3	1.6	9.4	8.8	1.3	5.0	3.5
15	8.3		○			4.3	6.4	1.8	8.1	8.4	0.2	2.5	8.2	7.8	0.4	5.1	3.4
16	7.7		○			10.0	1.4	0.0	8.4	6.1	0.0	1.9	10.0	7.6	3.7	5.2	3.9
17	1.3					7.6	6.7	1.6	5.8	2.7	1.6	1.9	7.4	8.0	8.4	4.8	3.0
18	7.1		○			6.2	7.1	8.6	0.8	2.9	1.8	3.3	1.7	2.5	7.4	4.5	2.8
19	8.5		○			0.5	0.6	0.4	0.7	0.5	0.4	0.6	0.6	0.5	0.5	1.3	2.4
20	9.1	○	○			9.3	1.3	0.5	7.3	9.1	0.4	9.4	9.5	8.8	0.8	6.0	4.2
Mean	6.8					6.5	4.2	1.8	6.0	4.9	1.2	3.5	7.1	7.0	3.7	4.8	3.2
Standard deviation	1.9					3.0	2.6	2.5	3.0	3.1	0.8	2.8	3.1	2.8	2.8	1.2	0.6

C

ID	Q1	Timing				Q3										Mean	Standard deviation
	Shittori	1	2	3	4	Soft	Warm	Rough	Sticky	Moist	Hard	Cold	Smooth	Slippery	Dry		
1	0.0		○			2.5	0.0	8.0	0.0	0.0	0.0	2.2	0.0	1.3	10.0	2.2	3.5
2	3.4					8.8	7.3	7.3	1.7	1.5	1.8	0.0	6.3	2.9	6.5	4.3	3.0
3	4.0					7.2	6.8	7.4	7.8	1.3	0.5	0.7	4.1	7.4	8.5	5.1	3.1
4	7.6				○	3.9	3.8	7.9	3.7	7.8	7.2	0.0	6.3	6.6	0.0	5.0	2.9
5	6.5					5.3	8.4	1.4	2.4	2.0	3.9	1.3	8.3	3.0	6.7	4.5	2.7
6	9.4	○				9.0	3.2	0.7	7.6	9.4	0.8	2.6	9.4	9.4	0.8	5.7	4.0
7	8.7		○			10.0	8.2	1.1	9.1	3.6	0.8	0.8	1.0	2.4	4.4	4.6	3.7
8	5.9					3.9	4.1	3.4	5.4	6.2	2.9	2.1	7.1	6.3	3.8	4.6	1.6
9	7.4		○			7.8	0.0	0.0	3.9	8.1	6.3	8.9	6.9	3.6	8.7	5.6	3.3
10	6.1					8.6	6.0	2.2	3.2	6.0	2.3	1.7	8.8	6.9	5.4	5.2	2.5
11	7.3		○			7.5	5.2	5.0	6.9	7.8	3.2	5.5	5.9	5.9	1.4	5.6	1.9
12	5.7					8.2	5.4	3.2	6.1	3.4	6.0	5.3	4.1	2.7	3.5	4.9	1.6
13	2.9					7.9	6.6	2.1	2.6	2.1	1.9	3.4	7.7	7.3	7.0	4.7	2.6
14	6.0					7.9	6.1	2.6	0.0	0.9	0.8	1.0	7.7	7.2	6.3	4.2	3.1
15	1.8					1.3	5.2	8.5	0.8	4.6	7.7	3.2	7.8	5.8	7.4	4.9	2.8
16	2.0					6.0	0.0	4.8	2.0	3.1	5.4	3.2	5.4	4.6	2.9	3.6	1.8
17	6.6					7.2	1.9	6.4	0.8	5.5	3.5	6.3	7.2	6.4	5.3	5.2	2.2
18	8.7		○			5.1	7.3	0.3	2.1	1.7	0.4	0.8	6.3	2.8	2.3	3.4	2.9
19	3.5					7.9	5.7	5.3	3.3	3.7	4.6	2.3	3.1	3.7	4.0	4.3	1.5
20	9.7	○	○			9.6	9.4	3.2	6.8	9.4	0.6	7.4	9.3	7.8	1.2	6.8	3.5
Mean	5.7					6.8	5.0	4.0	3.8	4.4	3.0	2.9	6.1	5.2	4.8	4.7	2.8
Standard deviation	2.7					2.4	2.8	2.8	2.8	3.0	2.5	2.5	2.6	2.3	2.8	0.8	0.7

D

ID	Q1	Timing				Q3										Mean	Standard deviation
	Shittori	1	2	3	4	Soft	Warm	Rough	Sticky	Moist	Hard	Cold	Smooth	Slippery	Dry		
1	0.0					1.6	2.0	6.5	0.0	0.0	3.2	1.5	2.2	2.5	10.0	2.7	3.1
2	6.5					3.5	6.8	3.3	5.0	4.6	7.3	0.5	7.7	5.2	5.1	5.0	2.1
3	2.5					7.8	7.1	1.0	7.1	0.3	3.5	0.7	2.4	0.6	9.6	3.9	3.4
4	5.0					4.8	2.5	2.2	4.2	2.9	6.6	2.9	6.8	6.2	5.0	4.5	1.7
5	2.3					6.8	7.7	0.3	4.0	2.8	3.4	0.4	8.6	9.0	8.7	4.9	3.3
6	8.9		○			7.1	0.8	0.7	6.7	4.6	0.7	0.6	5.6	6.8	0.9	3.9	3.2
7	6.8					10.0	10.0	7.8	8.6	2.7	0.8	2.0	0.0	8.6	3.3	5.5	3.8
8	5.4					3.4	5.0	2.9	3.3	5.6	6.1	5.0	3.5	6.0	5.8	4.7	1.2
9	1.1					6.1	2.5	2.0	0.0	4.6	3.8	1.3	8.6	2.0	7.3	3.6	2.8
10	7.0		○			8.1	6.2	2.1	2.8	6.4	2.7	3.1	7.1	8.4	3.4	5.2	2.4
11	6.2					6.2	5.9	6.9	5.9	6.9	3.6	3.4	5.3	1.9	2.8	5.0	1.8
12	3.6					6.6	2.1	3.6	3.6	2.1	2.4	4.0	1.5	1.1	6.9	3.4	1.9
13	3.9					5.7	2.3	1.5	4.6	2.7	3.6	2.7	8.2	6.8	6.7	4.4	2.2
14	8.4	○	○			8.2	9.5	0.2	0.6	7.4	1.5	1.1	8.4	8.2	5.5	5.4	3.7
15	7.5		○			5.8	6.1	0.9	0.6	1.3	3.3	0.7	7.3	5.7	7.2	4.2	2.9
16	3.6					5.9	0.0	4.9	0.0	4.4	4.1	7.4	5.6	6.3	6.4	4.4	2.4
17	4.8					3.4	3.1	1.1	0.9	2.9	7.5	6.4	8.2	6.8	5.5	4.6	2.5
18	6.2					9.5	7.4	0.3	5.8	4.2	0.6	0.9	5.7	1.9	4.9	4.3	3.0
19	2.3					9.4	9.0	4.0	0.7	4.4	1.1	0.8	3.2	3.5	1.9	3.7	3.0
20	9.3	○	○			8.5	1.0	0.8	1.4	9.1	1.1	1.1	9.2	6.9	0.9	4.5	4.0
Mean	5.1					6.4	4.9	2.7	3.3	4.0	3.3	2.3	5.8	5.2	5.4	4.4	2.6
Standard deviation	2.6					2.3	3.1	2.3	2.7	2.3	2.2	2.0	2.7	2.7	2.6	0.8	0.8

E

ID	Q1	Timing				Q3										Mean	Standard deviation
	Shittori	1	2	3	4	Soft	Warm	Rough	Sticky	Moist	Hard	Cold	Smooth	Slippery	Dry		
1	7.0		○			0.0	1.7	0.0	5.9	7.8	10.0	6.4	8.0	2.0	0.0	4.4	3.7
2	8.4		○			0.1	4.3	0.0	9.5	8.7	9.1	0.6	1.9	1.8	0.7	4.1	4.0
3	4.1					0.7	1.0	0.6	1.4	5.0	9.2	9.5	4.6	9.2	3.1	4.4	3.5
4	3.7					1.8	2.1	1.5	1.2	2.8	8.5	5.8	8.2	9.0	6.4	4.6	3.0
5	6.1					0.8	6.5	0.6	9.3	1.7	1.9	1.9	1.0	0.4	6.0	3.3	3.1
6	9.4	○				0.6	0.8	0.5	9.6	5.9	9.4	7.3	0.7	0.7	0.6	4.1	4.1
7	9.1		○			1.1	8.0	0.0	8.8	3.1	6.9	3.5	10.0	1.7	1.3	4.9	3.7
8	6.2					3.6	3.1	1.7	8.0	5.4	8.0	7.2	8.2	8.2	3.8	5.8	2.4
9	3.5					0.0	3.1	0.0	4.8	4.1	10.0	3.4	6.2	3.6	3.6	3.8	2.7
10	2.3					1.1	2.7	1.8	6.2	3.5	8.4	7.3	3.0	5.0	6.0	4.3	2.4
11	3.8					0.5	0.5	1.1	7.5	6.5	7.1	8.5	7.3	5.4	4.9	4.8	3.0
12	2.5					2.3	2.6	2.9	5.2	3.7	5.2	7.3	5.5	4.5	8.1	4.5	2.0
13	5.9					1.5	1.8	1.6	7.7	7.1	7.9	7.3	1.7	2.5	1.3	4.2	2.9
14	3.6					2.8	3.2	0.2	6.3	3.2	8.4	6.4	7.4	8.1	4.5	4.9	2.6
15	6.1					1.8	5.4	0.6	4.7	5.4	8.5	5.0	5.8	6.8	7.8	5.3	2.3
16	6.2					0.0	0.0	0.0	5.8	6.4	7.3	7.5	5.7	7.7	3.5	4.6	3.1
17	6.9					1.8	2.4	0.6	7.7	7.5	8.5	7.4	7.6	3.2	2.8	5.1	2.9
18	4.0					1.1	0.8	1.5	1.3	1.8	6.7	6.8	7.8	7.5	2.2	3.8	2.9
19	6.1					8.0	5.4	5.6	9.3	7.6	9.2	5.6	2.9	0.5	1.8	5.6	2.9
20	7.8		○			0.9	0.4	0.3	8.5	7.2	9.5	9.6	9.5	7.8	3.3	5.9	3.9
Mean	5.6					1.5	2.8	1.1	6.4	5.2	8.0	6.2	5.7	4.8	3.6	4.5	3.0
Standard deviation	2.1					1.8	2.2	1.3	2.7	2.1	1.9	2.3	2.9	3.1	2.4	0.6	0.7

F

ID	Q1	Timing				Q3										Mean	Standard deviation
	Shittori	1	2	3	4	Soft	Warm	Rough	Sticky	Moist	Hard	Cold	Smooth	Slippery	Dry		
1	7.5		○			0.0	0.0	0.0	8.2	7.2	8.2	2.6	10.0	6.9	2.9	4.9	3.8
2	7.8		○			0.0	0.0	0.0	7.3	7.2	7.1	6.2	6.4	2.0	2.2	4.2	3.3
3	0.4					0.5	0.4	0.4	0.5	1.0	9.8	1.7	3.2	9.3	1.0	2.6	3.6
4	6.8					5.1	0.9	2.0	1.1	6.1	5.8	9.3	7.2	7.7	3.3	5.0	2.8
5	0.9					1.3	6.2	0.5	9.7	0.7	9.7	5.3	1.5	3.1	9.3	4.4	3.8
6	9.1		○			0.6	0.6	0.7	9.3	8.3	9.3	6.5	0.6	1.4	0.6	4.3	4.1
7	7.4		○			2.3	1.3	0.0	6.6	2.7	8.4	9.1	10.0	5.6	3.1	5.1	3.4
8	5.6					3.4	3.9	1.2	6.5	4.6	3.1	3.0	6.4	5.8	3.9	4.3	1.6
9	6.6					0.0	0.0	0.1	0.0	3.2	2.7	8.3	8.1	10.0	5.4	4.0	3.8
10	3.9					1.7	1.5	1.2	6.3	3.9	7.2	6.9	3.4	3.1	5.5	4.1	2.2
11	6.1					1.5	1.8	1.1	7.7	8.5	8.8	4.9	2.7	3.1	2.0	4.4	2.9
12	5.9					2.4	1.4	2.9	6.9	5.2	7.6	9.2	7.7	6.4	6.8	5.7	2.5
13	2.4					0.7	1.2	0.7	6.8	6.1	8.0	7.7	3.7	6.9	4.2	4.4	2.8
14	5.1					2.1	5.5	2.1	6.9	5.3	8.5	3.5	8.2	7.7	4.6	5.4	2.3
15	4.0					7.5	4.8	1.3	4.1	6.3	7.6	7.0	1.9	3.9	5.2	4.9	2.1
16	5.6					0.0	0.1	0.0	4.8	5.0	7.4	7.7	8.3	6.0	2.7	4.3	3.2
17	0.9					0.4	0.5	0.2	0.6	0.3	9.4	8.3	9.5	9.7	0.6	3.7	4.4
18	1.9					0.5	0.5	1.3	0.5	1.6	7.8	8.4	8.3	8.1	6.2	4.1	3.6
19	6.2					2.1	1.3	4.1	0.7	9.2	6.9	8.1	1.0	2.2	3.6	4.1	3.0
20	7.7		○			0.5	0.6	0.6	6.7	8.4	9.4	9.3	9.5	7.3	2.2	5.7	3.8
Mean	5.1					1.6	1.6	1.0	5.1	5.0	7.6	6.7	5.9	5.8	3.8	4.5	3.0
Standard deviation	2.6					1.9	1.9	1.1	3.3	2.8	1.9	2.4	3.3	2.7	2.2	0.7	0.8

G

ID	Q1	Timing				Q3										Mean	Standard deviation
	Shittori	1	2	3	4	Soft	Warm	Rough	Sticky	Moist	Hard	Cold	Smooth	Slippery	Dry		
1	7.4		○			0.0	0.0	0.0	7.8	6.7	10.0	10.0	10.0	6.9	2.0	5.5	4.2
2	8.4		○			0.0	0.0	0.0	7.8	3.4	8.0	9.3	6.9	6.8	2.0	4.8	3.7
3	3.9					0.6	0.5	0.6	7.1	4.2	7.8	7.8	3.0	1.6	7.0	4.0	3.0
4	7.8	○				0.8	2.1	1.1	1.8	7.9	9.0	9.2	8.1	9.1	7.7	5.9	3.6
5	1.0					0.2	5.9	0.4	9.4	1.3	9.3	8.2	2.2	6.7	7.9	4.8	3.8
6	8.7	○				0.7	9.3	0.5	7.3	5.5	9.1	9.3	7.6	7.6	3.5	6.3	3.3
7	7.5		○			0.2	0.0	0.0	10.0	6.9	10.0	10.0	10.0	2.7	3.5	5.5	4.3
8	3.6					2.0	2.5	2.3	5.9	6.2	9.1	9.3	5.2	4.7	3.6	4.9	2.5
9	8.6		○			1.7	0.0	0.0	0.0	6.9	8.1	10.0	9.0	2.3	0.9	4.3	4.1
10	4.8					1.2	1.6	1.2	6.6	5.1	7.7	9.2	4.0	4.6	4.0	4.5	2.6
11	7.5		○			2.3	0.6	1.3	7.6	8.4	9.4	8.7	5.2	2.9	2.6	5.1	3.3
12	7.6		○			5.0	1.3	2.5	8.9	5.6	5.7	9.0	6.9	7.9	2.2	5.7	2.7
13	5.0					1.4	2.3	2.1	8.8	6.7	9.0	7.9	2.3	2.4	2.7	4.6	3.0
14	6.9					6.8	1.2	0.0	8.5	7.0	8.8	10.0	7.0	7.3	6.7	6.4	3.1
15	2.4					0.8	0.4	0.6	0.6	3.5	9.8	9.9	8.5	8.8	0.8	4.2	4.1
16	5.2					0.0	0.0	0.0	0.0	3.7	10.0	10.0	6.0	7.6	1.7	4.0	4.0
17	6.8					0.4	0.3	0.3	6.8	6.8	9.5	8.8	7.1	7.1	3.3	5.2	3.5
18	0.2					0.8	0.0	0.9	0.0	0.5	10.0	10.0	7.8	10.0	8.2	4.4	4.7
19	3.9					2.2	0.0	2.5	9.1	2.5	9.1	10.0	1.5	1.0	0.9	3.9	3.7
20	0.3					1.0	0.2	0.4	9.9	0.5	9.6	9.5	9.6	2.5	1.2	4.1	4.5
Mean	5.4					1.4	1.4	0.8	6.2	5.0	9.0	9.3	6.4	5.5	3.6	5.1	3.6
Standard deviation	2.8					1.7	2.3	0.9	3.6	2.4	1.1	0.7	2.7	2.8	2.5	0.7	0.6

H

ID	Q1	Timing				Q3										Mean	Standard deviation
	Shittori	1	2	3	4	Soft	Warm	Rough	Sticky	Moist	Hard	Cold	Smooth	Slippery	Dry		
1	1.4					0.0	0.0	0.0	1.6	0.6	10.0	10.0	10.0	9.0	7.9	4.6	4.7
2	6.9					0.0	0.0	0.9	7.1	7.2	8.4	10.0	8.9	5.5	0.8	5.1	3.9
3	1.7					0.7	1.1	0.6	0.4	0.8	9.5	9.6	7.6	9.7	2.1	4.0	4.1
4	2.6					1.9	0.0	1.0	1.0	1.1	6.5	9.4	8.8	8.5	8.2	4.5	3.8
5	0.7					0.6	0.4	0.5	9.7	0.5	9.6	9.8	8.7	2.7	9.4	4.8	4.5
6	7.1	○				1.5	0.7	0.5	7.9	5.5	9.3	9.2	5.3	0.5	0.7	4.4	3.7
7	7.7		○			0.0	0.0	0.0	9.1	3.6	10.0	10.0	10.0	1.6	0.7	4.8	4.5
8	3.3					0.8	0.6	0.2	3.0	0.7	9.8	9.3	0.4	3.5	5.6	3.4	3.5
9	6.4					0.0	0.0	0.0	0.0	2.3	10.0	10.0	10.0	9.9	0.0	4.4	4.8
10	3.8					3.1	0.6	1.8	2.4	4.1	8.5	9.0	5.8	5.2	6.2	4.6	2.7
11	3.8					0.7	0.9	1.1	0.8	1.0	9.0	9.6	9.6	9.6	9.5	5.1	4.3
12	7.0		○			2.7	1.6	3.1	6.1	4.7	5.7	8.9	8.0	8.2	3.3	5.4	2.5
13	2.5					1.3	0.6	1.5	3.4	1.9	8.6	9.5	1.0	3.8	3.4	3.4	3.0
14	4.1					1.0	0.9	1.1	1.5	3.4	8.0	9.2	6.6	8.2	3.8	4.3	3.2
15	6.4					0.4	0.7	0.7	0.9	8.8	8.4	9.4	6.7	8.3	2.1	4.8	3.8
16	6.2					2.8	0.0	0.0	0.0	2.5	6.1	10.0	10.0	10.0	4.0	4.7	4.0
17	3.5					1.7	0.4	3.1	0.8	2.1	0.4	8.8	7.0	6.7	7.4	3.8	3.1
18	1.7					0.5	0.0	0.6	2.9	0.3	8.5	9.1	6.6	9.3	3.7	3.9	3.8
19	2.0					1.9	0.2	2.6	8.6	5.8	8.1	9.8	1.9	1.1	2.1	4.0	3.4
20	1.4					0.6	0.8	0.5	2.7	2.7	9.2	9.3	9.5	7.4	8.5	4.8	3.9
Mean	4.0					1.1	0.5	1.0	3.5	3.0	8.2	9.5	7.1	6.4	4.5	4.5	3.8
Standard deviation	2.3					1.0	0.5	1.0	3.3	2.4	2.2	0.4	3.0	3.2	3.1	0.6	0.7

I

ID	Q1	Timing				Q3										Mean	Standard deviation
	Shittori	1	2	3	4	Soft	Warm	Rough	Sticky	Moist	Hard	Cold	Smooth	Slippery	Dry		
1	2.7					2.8	0.0	8.3	2.2	2.1	2.3	1.8	0.0	0.0	7.3	2.7	2.8
2	7.6		○			0.7	5.6	9.1	8.0	7.1	8.1	0.0	6.4	6.2	7.3	6.0	3.0
3	4.6					0.9	1.6	9.4	9.2	6.5	5.7	9.0	0.4	0.4	4.0	4.7	3.6
4	2.7					2.2	5.0	8.5	5.2	5.1	7.9	1.4	1.8	1.9	6.0	4.3	2.5
5	1.0					0.6	5.4	9.6	4.8	0.5	9.2	7.7	0.5	3.7	8.4	4.7	3.7
6	6.4					0.7	0.7	6.3	5.3	2.4	6.7	5.5	0.5	6.2	7.2	4.4	2.7
7	2.8					2.5	6.8	10.0	0.0	2.0	8.7	7.1	0.5	1.2	7.9	4.5	3.6
8	3.4					4.1	4.8	8.4	1.5	1.6	8.4	0.0	1.6	2.4	8.4	4.1	3.1
9	0.0					0.0	0.0	10.0	0.0	0.0	10.0	10.0	0.0	0.0	10.0	3.6	5.0
10	6.2					6.2	7.0	7.2	2.9	6.6	4.3	3.0	3.9	3.2	6.3	5.2	1.7
11	6.6					3.4	3.5	9.2	6.4	8.0	8.5	5.8	3.4	4.5	4.6	5.8	2.1
12	1.2					3.7	5.2	9.4	3.4	3.3	4.0	5.4	1.7	3.7	8.5	4.5	2.5
13	3.2					1.1	1.1	9.3	2.9	5.7	8.5	7.7	0.6	0.9	2.4	3.9	3.3
14	5.8					3.2	6.7	8.4	8.6	6.1	7.9	2.4	6.1	6.6	4.9	6.1	2.0
15	0.6					0.4	2.0	9.5	0.6	0.7	8.8	2.6	0.2	1.0	0.8	2.5	3.4
16	1.5					3.5	0.0	10.0	0.0	1.5	6.6	2.1	4.9	5.2	7.5	3.9	3.2
17	8.1	○	○			6.3	0.9	7.8	1.1	7.1	7.8	6.6	3.1	2.7	2.0	4.9	2.9
18	0.1					0.4	0.0	10.0	0.0	0.3	8.8	0.4	0.0	0.0	9.7	2.7	4.4
19	2.3					1.2	1.1	8.7	0.5	4.1	9.1	0.8	1.0	1.3	1.6	2.9	3.1
20	5.8					1.5	1.0	9.5	9.3	8.6	9.2	7.1	0.2	2.2	2.5	5.2	3.7
Mean	3.6					2.3	2.9	8.9	3.6	4.0	7.5	4.3	1.8	2.7	5.9	4.3	3.1
Standard deviation	2.6					1.9	2.6	1.0	3.3	2.8	2.0	3.2	2.1	2.2	2.8	1.1	0.8

J

ID	Q1	Timing				Q3										Mean	Standard deviation
	Shittori	1	2	3	4	Soft	Warm	Rough	Sticky	Moist	Hard	Cold	Smooth	Slippery	Dry		
1	0.0					0.0	0.0	10.0	0.0	0.0	8.4	1.6	0.0	1.8	10.0	2.9	4.3
2	6.7					0.0	7.4	9.3	7.1	5.7	7.5	9.6	3.8	3.6	4.3	5.9	2.8
3	3.7					1.4	0.5	9.5	7.3	6.6	8.1	7.8	0.5	1.6	3.4	4.6	3.4
4	0.0					0.3	0.9	10.0	0.9	0.6	10.0	9.5	0.9	0.8	9.6	4.0	4.6
5	0.6					0.3	5.3	9.2	8.6	0.3	9.2	1.7	0.6	0.3	8.1	4.0	4.0
6	0.6					0.8	1.8	9.2	0.8	0.9	9.2	1.8	0.9	0.8	6.5	3.0	3.5
7	0.1					3.0	0.0	10.0	1.0	2.5	8.0	2.7	0.2	0.0	9.2	3.3	3.9
8	2.0					3.4	7.7	7.3	4.8	3.7	3.6	1.4	2.4	3.0	7.3	4.2	2.2
9	0.0					0.0	0.0	8.8	0.0	0.0	8.7	8.5	0.0	0.0	10.0	3.3	4.6
10	4.4					2.5	2.4	7.9	7.3	5.8	7.5	4.8	2.8	3.5	6.9	5.1	2.1
11	6.2					1.8	1.1	8.8	6.2	6.9	6.1	7.9	1.2	0.9	3.8	4.6	3.0
12	1.8					6.9	2.7	7.6	6.9	3.1	4.0	3.2	1.4	3.9	6.2	4.3	2.2
13	6.6					2.0	1.1	9.4	7.1	3.1	9.3	8.5	1.1	1.6	2.3	4.7	3.4
14	6.5					8.0	1.7	8.4	1.5	6.2	7.0	2.1	7.5	7.5	2.6	5.4	2.8
15	0.2					0.2	2.3	9.4	0.4	0.4	9.6	0.8	0.0	1.6	7.0	2.9	3.8
16	1.3					0.0	0.0	10.0	3.2	2.1	10.0	6.5	3.2	3.4	4.4	4.0	3.5
17	6.2					3.5	3.8	9.0	1.0	7.1	7.5	4.7	0.8	0.5	3.4	4.3	2.9
18	4.6					6.0	1.4	8.6	9.7	7.8	6.5	0.1	0.9	2.0	0.3	4.4	3.6
19	1.6					0.5	0.6	9.5	1.6	2.9	9.5	8.6	1.6	1.5	3.3	3.7	3.6
20	7.2	○				0.6	1.5	9.6	7.5	8.3	9.3	8.1	1.4	0.4	2.0	5.1	3.8
Mean	3.0					2.1	2.1	9.1	4.1	3.7	8.0	5.0	1.6	1.9	5.5	4.2	3.4
Standard deviation	2.8					2.4	2.3	0.8	3.4	2.9	1.8	3.3	1.8	1.8	2.9	0.8	0.7

K

ID	Q1	Timing				Q3										Mean	Standard deviation
	Shittori	1	2	3	4	Soft	Warm	Rough	Sticky	Moist	Hard	Cold	Smooth	Slippery	Dry		
1	0.0					1.9	1.5	8.3	0.0	0.0	1.9	0.0	1.9	0.0	10.0	2.3	3.5
2	1.5					9.5	1.6	6.0	0.8	0.7	1.2	0.0	5.0	6.5	8.4	3.7	3.4
3	1.6					9.0	8.3	8.5	1.0	1.0	0.9	0.9	1.7	1.1	9.3	3.9	3.9
4	2.5					5.1	3.0	4.8	1.5	2.7	7.0	5.1	6.2	6.5	8.7	4.8	2.2
5	0.9					5.4	3.4	1.5	3.6	0.5	2.2	1.0	6.5	3.5	9.4	3.4	2.7
6	6.8					6.2	2.3	3.8	2.2	4.0	5.9	1.7	6.7	6.3	4.0	4.5	1.9
7	8.6	○				7.3	10.0	9.0	1.9	8.1	8.3	0.0	0.0	1.5	8.2	5.7	4.0
8	1.6					3.4	5.7	6.3	4.1	3.6	1.8	2.2	4.2	2.3	3.5	3.5	1.5
9	0.0					6.7	4.0	5.9	3.2	6.1	5.2	3.9	5.9	4.1	4.1	4.5	1.9
10	2.7					7.4	7.0	3.7	3.8	3.6	2.0	2.8	5.8	5.5	7.6	4.7	2.0
11	3.4					5.8	5.3	6.8	1.7	2.6	4.4	4.2	4.1	7.4	6.7	4.8	1.8
12	2.8					6.7	6.3	7.8	3.7	1.9	2.5	4.0	3.3	1.1	7.9	4.4	2.4
13	3.1					6.3	3.4	7.7	3.0	2.0	4.0	2.3	6.4	2.9	7.4	4.4	2.1
14	3.4					6.4	8.0	8.0	0.0	2.8	8.0	0.7	2.9	1.5	9.1	4.6	3.3
15	1.0					8.0	6.4	7.4	0.8	1.3	7.2	9.4	5.8	1.0	0.9	4.5	3.4
16	2.7					7.8	0.0	5.1	1.2	1.7	2.3	1.7	5.9	5.1	6.0	3.6	2.5
17	1.9					6.6	2.2	6.9	1.7	1.3	2.8	5.8	8.3	1.7	8.4	4.3	2.9
18	4.1					3.0	6.9	7.3	1.7	0.9	5.6	0.6	1.0	1.7	8.9	3.8	3.0
19	2.1					6.4	8.1	7.5	1.1	1.2	6.2	2.6	6.8	6.4	9.4	5.3	2.9
20	6.6					7.9	8.7	9.3	0.2	2.4	1.6	7.5	1.5	5.6	5.0	5.1	3.2
Mean	2.9					6.3	5.1	6.6	1.9	2.4	4.1	2.8	4.5	3.6	7.1	4.3	2.7
Standard deviation	2.2					1.9	2.8	2.0	1.3	2.0	2.4	2.6	2.3	2.4	2.5	0.8	0.8

L

ID	Q1	Timing				Q3										Mean	Standard deviation
	Shittori	1	2	3	4	Soft	Warm	Rough	Sticky	Moist	Hard	Cold	Smooth	Slippery	Dry		
1	0.0					6.3	1.5	8.4	0.0	0.0	3.7	1.4	0.0	1.4	10.0	3.0	3.6
2	1.3					7.8	7.9	9.5	0.0	0.0	0.0	0.0	0.8	2.2	8.3	3.4	4.0
3	1.9					8.7	8.5	9.2	2.8	1.3	1.9	0.5	1.3	3.7	9.5	4.5	3.7
4	0.0					3.5	2.1	8.0	0.9	0.9	7.9	7.3	0.5	0.9	9.2	3.7	3.6
5	0.9					7.7	8.1	0.3	0.4	0.3	2.0	2.7	9.3	9.4	7.9	4.5	4.0
6	2.1					3.7	1.1	7.8	1.2	1.5	6.0	1.5	2.0	4.8	6.0	3.4	2.4
7	0.8					7.6	9.2	10.0	2.0	1.8	2.1	1.6	1.4	0.1	8.5	4.1	3.8
8	2.4					3.2	5.7	5.9	1.6	2.0	3.4	2.8	4.1	4.8	7.0	3.9	1.8
9	0.0					1.3	0.0	8.9	0.1	0.0	8.3	0.0	0.0	0.0	10.0	2.6	4.2
10	3.6					7.5	3.4	5.7	1.9	3.8	2.8	2.7	7.8	6.1	7.2	4.8	2.1
11	2.4					7.0	5.9	6.9	2.2	4.2	2.9	2.0	4.4	4.0	2.9	4.1	1.8
12	4.2					8.1	5.8	6.8	3.0	3.1	2.1	2.4	2.8	5.6	8.1	4.7	2.3
13	2.4					3.8	3.7	8.9	3.5	1.0	6.1	1.6	3.5	1.2	8.7	4.0	2.8
14	2.6					9.2	8.0	2.2	0.0	2.5	6.2	1.1	9.2	8.1	9.3	5.3	3.6
15	0.2					4.6	6.6	9.0	0.9	0.6	5.1	0.7	0.5	1.3	0.8	2.8	3.0
16	2.4					4.0	1.4	7.7	2.5	1.5	7.3	2.4	3.7	5.5	7.7	4.2	2.5
17	0.4					8.6	4.4	9.2	0.5	0.3	1.3	1.9	2.5	0.9	0.3	2.8	3.3
18	5.1					8.9	5.8	6.3	0.6	2.9	0.7	1.0	1.1	0.4	9.2	3.8	3.4
19	0.3					5.4	4.8	9.5	0.3	8.6	4.5	3.5	3.4	3.4	1.5	4.1	3.0
20	3.7					3.1	8.5	9.3	2.8	2.4	2.1	0.7	1.5	4.2	6.5	4.1	2.8
Mean	1.8					6.0	5.1	7.5	1.4	1.9	3.8	1.9	3.0	3.4	6.9	3.9	3.1
Standard deviation	1.5					2.4	2.8	2.5	1.2	2.0	2.5	1.6	2.8	2.7	3.1	0.8	0.9

Table S2 Sensory scores of 12 materials. Q2: “Why did you feel that (*Shittori* feel)?”

A

ID	Comment
1	指につく感じがかなりして、水分を含んでいるような粉だった。
2	粉から指を離してもずっとくっついている感じがしたから。
3	粒が細かいような気がした。なめらかだった為。
4	指先で触った時に、指になじむ感じがした。
5	べたつく。ぬりひろげると、ややしっとりする。
6	少し指にまとわり付く感じがした。
7	指になじんでいく感じがしたから。
8	ちょっと冷たくてベタついたように感じた。すりこまれるかんじ。
9	手にくっつく感じがしたから。
10	やわらかさを感じたから。
11	くっついてくる、まとわりつく。
12	肌にくっつく感じがしたため。
13	手にすいつく感じがしたから。
14	粉が細かく粒感を感じないから。
15	粘性を感じる。
16	粉に重みがあったため。
17	さらさらしたけど少し湿っている感じがした。
18	ぬるっとしてた。
19	水気はあるが、あまり手になじまない感じ。触った瞬間はちょっとしっとり感。
20	手につきやすかったから。

B

ID	Comment
1	さらさらしているが、指にはりつくような感じがした。
2	サンプルを触っている時に、粉が指に付いたままだったから。
3	しっとりというよりは、ふわふわしていた気がした。
4	滑らかな感じがして、しっとりって感じではなかった。
5	粉から液体になった時に指がしっとりしたから。
6	しっとりなめらかな感じがしたけど、サラサラしているようにも感じた。細やか。
7	触った瞬間だけギシッとして、片くり粉みたいで、触ってるときは、すごくいいフェイスパウダーみたいにしっとり。
8	つめたいものが指のすきまに入ってきたような感じだった。
9	つぶつぶ感が無かった。
10	さらさらとした感じがやわらかだったから。
11	滑るのにくっついてくる不思議なかんじ。
12	肌にくっつく感じがしたため。
13	手にすいつく感じがした。
14	さらっと汗を吸ってくれるような感じがした。
15	指にくっついてくるから。
16	指にくっつく感じがしたため。
17	さらさらだった。スムーズに指を動かせた。
18	つまんだときにザラッとした感じを感じなかったから。
19	手になじむ感じがしたから。
20	なめらか、手に適度にくっつく。

C

ID	Comment
1	毛ばだっている感じがした。乾燥していると感じた。
2	表面がふわふわしていたから。
3	前のものよりは吸いつきを感じた。
4	ぬるぬるした感じがしたから。
5	押している中心はしっとりしていた（周囲はやわらかい毛の感触）
6	指に少しくっつく感じがした。
7	触ったときに指に吸いつくような、やわらかいような感じがしたから。
8	パサパサしてなかた。
9	キメ細かい感じがしたから。
10	すべすべとしたやわらかさを感じたから。
11	ちょっとくっつくかんじがするから。
12	ざらざらしたため。
13	さらさらしてた。
14	毛が柔らかかったので滑らかさを感じたが、ややパサツキを感じた。
15	硬くて、滑らかい。
16	少しざらつきを感じたため。
17	すべりすぎず、ざらざらしすぎず。
18	ぬるっとした布のような素材でさわりやすかったから。
19	触った瞬間は少しザラついているが、動かしていると少し手になじみ、しっとりとした印象を持った。
20	なめらかだった。

D

ID	Comment
1	細かいざらざらで乾燥している感じがした。
2	そんなにざらざらしていなかったから。
3	短い毛が凝縮した様だった為。硬めのフリース生地の様だった。
4	触ったサンプルの中で一番特徴がつかめなかった気がします。
5	しっとりというよりは滑らか（すべすべ）。
6	指との密着感。
7	ふわふわしているから。
8	全く乾いているという感じではなかった。
9	粗さはないけど、滑る感じがしなかった。
10	すべすべとしたやわらかさを感じたから。
11	(キメが細かい）布をさわっているような感じ。
12	滑りにくかったため。
13	さらさらしてた。
14	素材がクッションのように感じた。程よいなめらかさ。
15	スポンジの硬い版。
16	ヒトの皮膚のような感じでした。
17	しっとりしていると言われればしっとりしているし、していないと言われればしていない。ぬれている感じはしない。
18	手に吸いついてくる感じがあったから。
19	少しあたたかさがあり、乾いた感じ。
20	なめらか、毛足が短い。

E

ID	Comment
1	つるつるで、湿った感じがかなりした。
2	指とサンプルがくっついている感じがしたから。
3	指を置いた時に冷たさと少ししっとりした感じがした。
4	どちらかというとスベスベした感じがしたから。
5	指がひっぱられすぎるが、水っぽさがある。
6	指がくっつくとしっとりしているように感じる。
7	２つめ（アルミニウム）のと同じで、指の油で吸いつく感じがしたから。
8	少し弾力があるような、硬いゲルみたいだった。自分の手の湿り気かも？
9	少しつっぱる感じがしたから。
10	かたさが強く感じられたから。
11	ガラスをさわっている感じ。
12	まさつを感じたから。
13	湿った感じがしたから。
14	かたいけれど乾きは強く感じなかった。
15	アクリル板みたい。
16	指を滑らせるときに、滑りやすく感じたため。
17	指のすべりがあまりよくなかった。
18	ぬるっとした感じはじゃっかんあったが、つるっとしすぎているから。
19	しっとりというよりは少しベタついている印象。
20	くっついた感じがした。

F

ID	Comment
1	指は動かせるが指がくっつく感じがした。
2	表面にでこぼこがなかったから。
3	吸いつく感じがしなかった。硬めだった。
4	さっきのサンプルよりも少ししっとりした感じがあったから。（サンプルに指をのせたとき）
5	水っぽさがない、乾いている。
6	指にくっつく感じ。
7	4つめと同じにしか思えませんでした。
8	少し樹脂のように弾力を感じた。
9	冷たくて滑らかだったから。
10	かたいように感じたから。
11	すべりがわるくて、湿っているかんじ。
12	冷たさと同時にしっとりした気がした。
13	しっとりというよりはつるつるした感じ。
14	冷たさはあまり感じなかったが固かったから。
15	きゅっきゅっとしていた。
16	指を滑らせやすかったため。
17	つるつるしていた。
18	ザラザラしていたように感じたから。
19	少しベタついて指にまとわりつく感じ。湿っぽい感じで少ししっとりしている印象。
20	くっついてくる感じ（動かしてる時）。なめらかだけど滑りにくさが多少あった。

G

ID	Comment
1	冷たい感じがして、指にはりつく感じがあった。
2	サンプルと指のくっつきが大きかったから。
3	なめらかではなく、摩擦が強い。
4	さっきのサンプルよりもまたさらに湿り気を感じたから。
5	引っ張られる力が強い。
6	指にくっつく感じがした。
7	サンプル自体のしっとりさというより、指の油でしっとりする感じ。
8	冷たく乾燥していた。冷たいので表面に水分がついているのかな？と感じてしまう。
9	ひんやりしたから。
10	冷たく感じたせいか、一つ前のサンプルよりはしっとりしていると思った。
11	少し滑りが悪く感じたので。
12	肌にくっつく感じがした。ひんやり感もあった。
13	つるつるしてた。窓ガラスみたい。
14	少し濡れたような感じがした。
15	金属。
16	冷たく感じたため。
17	つるつるしていたが、指のすべりがあまりよくなかった。
18	つるっとしたから。
19	冷たいが、手にまとわりつく。少しベタッとした感じがした。
20	何もなかった。つるつるした表面だったから。

H

ID	Comment
1	さわっていて、指にはりつくこともなく、滑らかだった。硬さを感じた。
2	表面がつるつるに感じたから。
3	ツルツルして、硬い感じがした為。
4	しっとりというよりもサラサラで滑る感じがしたから。
5	水っぽさがほとんどなかった。
6	触った瞬間指がピタッとくっついた。
7	指にくっついてくる感じがしたから。
8	硬くて冷えていたので乾燥したイメージだった。
9	ひんやりしていたから。
10	冷たさとかたさを感じたから。
11	さらさらしている。
12	すべりここちが良かったため。
13	ひっかかる感じだった。
14	柔らかさがなかったから。
15	台所（アルミ製ぽい）。
16	ひんやりしていたため。
17	サンプルが乾いていた感じがしたから。
18	冷たくて、ひっかかるところがあり、金属感が強かったから。
19	冷たすぎて手になじむ感じもあまりなかったから。
20	するする滑るけど、くっつく感じがあまりなかったから。

I

ID	Comment
1	ガサガサだったが、さわっていくうちに湿っぽさを感じた。
2	指を動かすときに少し抵抗がある気がしたから。
3	乾いているとは感じなかったが、冷たく、吸いつく様な感覚だった。
4	表面がザラザラしているけど、指が少しサンプルになじむ？感じがした。
5	水っぽさがない。やわらかさがない。
6	触った瞬間は指がくっついていたのでしっとり感を感じたが、こすり始めたらザラザラしていてしっとり感は全く感じなかった。
7	ざらざらしていて、くっつく感じもなかったから。
8	触れた後指が元と同じように感じたから。
9	ざらざらしていたから。
10	あまりかたさを感じなかったから。
11	ざらざらしているのに、なぜかしっとりしているような気が。まさつ？
12	粗さが強かったため。
13	ざらざらしていた。
14	なめらかな凸凹に柔らかさを感じたから。
15	ざらざらして、ゴムみたいだった。
16	ざらざらしていた。
17	少しだけ湿っている感じがした。
18	ざらざらしてたから。
19	目が粗いが、冷たく少し湿っている感じがしたから。
20	ざらざらしていたけど、くっつく感じがしたから。

J

ID	Comment
1	ザラザラ、ガサガサとしていた。やすりみたいだった。 水分のある感じがしなかった。
2	少し指を動かしづらかったから。
3	ザラついていてしっとりしていなかった。
4	表面がザラザラしてて、とってもかたかった。
5	ザラザラ、硬い。
6	ザラザラしていた。
7	ざらざらしているから。
8	ざらついて、すこし粉っぽかった。
9	ザラザラしていたから。
10	ざらざらとした感じがしたから。
11	粗いのに少し湿っているかんじ。粗くなかったら、もっとしっとりに感じそう。
12	ざらざらしていた。
13	しめった感じがした。
14	やや肌に吸い付く感じがあった。
15	硬くてざらざら。
16	ざらつきを感じたため。
17	ざらざらしていて、すべりがあまりよくなかったから。
18	でこぼこしており、少し痛いように思ったが吸いつくかんじがあった。
19	目が粗く、ザラザラして手につかない。
20	ざらついてたけど、ぺとぺとした。

K

ID	Comment
1	触りごこちが乾燥しているように感じた
2	表面がふわふわだったから。
3	表面が乾いていて、綿の様だった為。
4	サンプルの表面が乾いてて、指がすいつく感じがあまりしなかったので、しっとり感はさほど感じなかった。
5	さらさら感が強い。
6	一方向にこすった時はしっとり。その逆方向にこすった時はあまり感じなかった。
7	指を置いた瞬間だけしっとり感じました。
8	ざらざらしていた。
9	ふわふわしているだけだから。
10	さらさらとしてかわいた感じがしたから。
11	手ざわりはどちらかというとさらさら。
12	少し粗さを感じたため。
13	さらさらした感じでした。
14	パサツキを感じた。
15	少ししっとりした毛皮。
16	布のように感じたため。
17	ざらざらはしていたが指がなめらかに動いた。
18	あたたかさはあったが乾いており、カサカサしていたから、ちょっとかたいように思ったから。
19	乾いていて温度はあまり気にならなくとも、手にはなじまなかった。
20	やわらかかった。

L

ID	Comment
1	さわった瞬間、カサカサしていた。
2	サンプルの表面がザラザラの布のように感じたから。
3	綿毛のような触り心地でしっとりは全く感じなかった。
4	しっとりというよりザラザラしている感じがしたから。
5	滑らかすぎ、水っぽくない。
6	しっとりというよりはふさふさしていた。
7	さらさらしていたから？
8	さらっとしていた。
9	ガサガサした感じがしたから。
10	かわいた感じがしたから。
11	くっつかなかったので。
12	ちょっと粗さを感じたため。
13	ざりざりしてた。
14	乾いた感じだったから、毛流れと逆だと余計にパサツキを感じた。
15	毛皮。
16	布のような感じがしたため。
17	乾いた生地っぽい。
18	ザラザラしていたがもふもふしたものが手につく感じがしたから。
19	ザラザラしてあまり手につかない、なじまない感じがしたから。
20	触った瞬間はしっとりしたが動かすと感じなくなった。

Table S3 Sensory scores of 12 materials. Q4: the subjects freely described the tactile feel of the materials.

A

ID	Comment
1	ふつうの粉よりもしっとりしている感じがした。
2	１つめの粉よりももっと粒が細かくて、片栗粉みたいだなあと思いました。
3	触り心地が良かった。なめらかで指につく感じがした。
4	こういうパウダーのファンデーションがあったらいいなと思った。
5	だまになる。べたつく。
6	きもち良かった！最初触っている感じがあまりしなかった。
7	動かすうちに、指の中でまとまっていくような感じがしました。
8	前の粉よりベタついていた気がした。
9	少し「もちっ」とした。
10	やわらかい感じがした。あつみを感じた。
11	
12	一瞬粉だとわからないくらいはなめらかだった。
13	指を擦り合わせるのがスムーズでした。
14	
15	不思議な感触だった。
16	猫の毛のようでした。
17	だんだん温かくなっていった。湿っている感じがしてしっとりしていると思った。
18	前のこなと同じように感じた。
19	粘り気、水気がある感じ。キメが細かく、やわらかい印象。粘り気のせいか滑りやすいとはあまり感じなかった。
20	ベビーパウダーのようなやさしさを感じた。

B

ID	Comment
1	シルクみたいなさわりごこちだった。
2	粒の大きさがとても小さくて、すべすべでした。
3	ふわふわとしていて、きめ細かい粒子だと思った。おしろいを触っているようだった。
4	前のサンプルに比べてしっとり感とか湿り気はあんまりなかった。
5	粉をこする毎にしっとり、なめらかになった。
6	シルクのようななめらかさ。ぬるぬるしているような、サラサラしているような。しっとりはしているけどすごく滑りが良いと感じた。
7	気持ちよかったです。
8	さらさらして触りごこちがよかった。シルク布のようなかんじ。
9	片栗粉みたいだった。手にくっつく感じがした。
10	細かい粉を触っているような感じがして、さらさらとしていた。
11	
12	肌になじむ感じがした。
13	しっとりしてて、さらさらもしてると感じました。さわった感じ不快感ゼロでした。
14	ベビーパウダーのような感じ。さらさらしっとり。
15	片栗粉
16	すごくやわらかかったです。
17	さらさらでなめらか。温度は何も感じないくらい。
18	つるつるの布を触っているみたいに感じた。
19	ふわふわしている印象。キメが細かい？手ざわりが良くて好き。手になじみやすい。
20	粒子が細かい感じ。やさしさ感じた。

C

ID	Comment
1	さわった瞬間も、さわってからもずっと毛ばだっていてしっとり感はなかった。
2	指を置いたときにふわっとしている感じがしました。
3	上質な繊維みたいだった。なめらかではなかった。
4	表面が少しでこぼこしてるように感じた。
5	熱がこもりやすい。
6	ツルツル。ケータイの画面をふく布みたいと感じた。繊維が細かそう。
7	むずかしいですね。
8	高そうなセーター。ふわふわしていた。
9	ひんやりした。触っていて気持ち良かった。
10	すべすべとしていてなめらかだった。やわらかさを感じた。
11	
12	粗さを感じたが、触り心地はよかった。
13	さらさらしてました。
14	程よい温かさを感じた。
15	気もちよくない。
16	カーペットの触感に似ていました。
17	少しだけざらざらした感じ。なめらかなざらざら？
18	触り心地がよかった。ずっと触れる。
19	少し粗い。布テープとか、包帯とか布繊維系のものを触った感触に似ている。指を動かしていると、少し温かくなったように感じた。
20	皮のような感じ。なめらか。こういう服持っている。最近はやってる。好き。

D	
ID	Comment
1	無機物でも人の肌でも布でもなく… 不思議なさわりごこちだった。
2	凸凹が少しあって、指を動かしやすかった。
3	黒板消しの消す面をなでている様だった。しっとりさは感じなかった。
4	やわらかさとか湿感とかが触ったサンプルの中で中間にあるような気がした。
5	クリームをぬった後の肌をさわった時に近い。少し毛が気になる。
6	しっとりだけどすべすべしている感じ。自分が持っているアンゴラのコートの肌触りと少し似ていた。
7	折り返したときにくっついてくる感じ。
8	温かくも冷たくもなかった。細かい感じがした。メガネふきみたい。
9	不思議。粗さは無く滑らかなのにしっとり感を感じなかった。
10	すべすべとしてなめらかであった。
11	
12	摩擦を感じて、滑らかさがすくなかった。
13	ファンデーションの表面みたいでした。
14	柔らかい皮のような感じ。
15	低反発マットレスの硬い版。
16	少しかたいヒトのひふを触っているようでした。
17	きめ細かいと感じた。指がまあまあなめらかに動く。
18	パサパサしていなくまとまりがあるように感じた。
19	触った瞬間から温かい。少し目は粗いが、不快に思わない程度。
20	良い。動かすとよりしっとり感じた。

E	
ID	Comment
1	つるつるだったけど、そこまで滑らなかった。指のひっかかりがかなりあった。
2	表面に全く凸凹がなく、つるつるに感じた。
3	硬く、表面がツルツルしていた。水をはじく感じがした。
4	かたかった。
5	指が動かしにくい。ひっぱられる。
6	アクリル板とかガラスを触っているような感じ。指がくっついてこすりづらかった。
7	2つめより厚み？重み？温かさ？みたいなのを感じました。
8	タイルみたいな手触りだった。樹脂のようなかんじ。
9	少しつっぱる感じがした。プラスチック板を触っているような感じがしたから。
10	つるつるとしていてかたく感じた。滑らかではなかった。
11	ガラスみたい。
12	冷たく感じた。
13	指を動かすのに少し力が必要だった。
14	つるつる。少し抵抗がある。
15	スマホみたい。
16	ガラスよりも、少し滑りやすい感じがしました。
17	サンプルはつるつるしていたけど、指のすべりがそこまで良くない。
18	動かした瞬間ぬるっとした触り心地だった。
19	ベタッとして滑りにくい感じ。
20	冷たい。手がすべりにくかった。

F	
ID	Comment
1	つるつるとして硬さもあったが、指にはりつきがあった。
2	表面がつるつるで、冷たさや温かさはあまり感じなかったです。
3	硬く、ツルツルしていると思った。
4	冷たかった。表面がスベスベしてたけど1つ前のサンプルよりも湿り気があったように思った。
5	多く触った所は温かく、あまり触れていない所は冷たい。平らなプラスチックを触った感じ。
6	指がくっつきすぎて、こすりづらかった。しっとり感はすごく感じた。
7	4つめと同じですね、これは。
8	ガラスのような樹脂のようなかんじ。でもすごく冷たくは感じなかった。
9	一番滑る感じがした。冷たさは3番目くらい。
10	かたさが感じられ、指にひっかかる感じがした。
11	
12	ひんやりしていて、滑らかだった。
13	ガラスやアクリル板みたいな感じ。
14	少し摩擦を感じた。ひっかかりがあった。
15	ほどよい温感。
16	指を滑らせるときに、少し湿っているように感じました。
17	つるつるしていてかたい。一瞬何もさわっていないかと思った。
18	多少ザラついていたことが印象に残った。
19	指に触ったところがまとわりつく感じで、湿ったベタついた印象。最初のサンプルに似ている。すべりにくく温度もない。
20	すべりにくかった。

G

ID	Comment
1	冷たい金属をさわっている感じだった。
2	指を置いたとき、ひんやりしました。
3	指にくっつく印象が強かった。
4	冷たくて、かたかった。サラサラ、滑る感じがしたのは前のサンプルと一緒だけど、それに湿り気が加わった感じがした。
5	ガラスを触った感じ。指を反対方向に移動する時につっかかる、ひっぱられる。
6	触った瞬間はくっつく感じがしたけど、こすり始めると思ってたよりサラサラしているというか、すべりが良いなと感じた。くっつく＝湿っていると感じる。
7	プラスチックとか、金属みたい。
8	最初の方のサンプルとにていた。ガラスのようなかんじ。窓ガラスのようなかんじで、車のガラスより硬く感じた。
9	冷たくて気持ちが良かった。滑らかだけど少しだけつっぱる感じがあった。
10	冷たさを強く感じた。
11	１つ前のサンプルよりしっとり。
12	肌にフィットして、とても滑らかだった。
13	指が動かしにくかった。
14	つるつる。ぺたぺた。
15	ひんやりしていて気持ちいい。
16	ガラスを触っているような感じがしました。
17	冷たかった。表面が平らな感じ。つるつるしている。
18	かがみを触っている気分だった。
19	冷たい。ベタッとして少し水気はあるが滑りは悪い。表面が粗い印象はなかった。指を動かしにくく、手にまとわりつく感じ。
20	つるつるしていた。

H

ID	Comment
1	さわっていて引っかかりがほとんどなかったように思う。
2	指を置いたとき、冷たいと思いました。少しだけ凸凹があるように感じました。
3	表面は冷たくツルツルしていて指に吸いつく感覚はなかった。触った瞬間“冷たい”と感じた。
4	冷たかった。摩擦をあんまり感じなかった。サラサラしてた。スベスベ。
5	ガラスを触ったような感覚。
6	冷たくてきもち良かった。指がはりつく感じがしたが、少し滑らかさも感じた。金属を触っているような感じ。
7	金属みたい冷たかったです。
8	ガラスのような無機質なかんじ。
9	金属板を触っているような感じがした。つめたい。
10	非常に冷たく感じた。かたさは感じたが、この前のいくつかのサンプルよりもなめらかであるようには感じた。
11	さらさらしていて気持ちいい。
12	ひんやりとしていた。
13	冷たかった。指が動かしにくかった。
14	冷たくてつるつるしていた。ガラス板のような感じ。
15	冷たくて気持ちよかった。
16	冷たく、滑りやすかったです。
17	冷たい、まあまあなめらか。指を動かしやすい。
18	金属のような印象が強かった。
19	冷蔵庫で冷やしたのではないかと思うほどの冷たさ。第一印象冷たい。目は粗くなく、むしろツルツルしている印象だが滑りは悪い。固く、温度がない。
20	冷たい。ひんやりしていた。滑らかだった。

I

ID	Comment
1	さわった瞬間はブツブツしていた。さわっていてもやっぱりざらざらとしていた。
2	１つ前のサンプルよりでこぼこ感が少なかった。
3	ザラザラしていた。指をスライドしにくかった。
4	表面がザラザラしていた。
5	ザラついている。こするごとにだんだんと熱がこもる（指からの熱がうつった？）。
6	ザラザラしているけど、キメ細かい感じがした。ただ滑らかさは全く感じなかった。
7	キムワイプをかたくしたみたいなざらざら感。
8	布のような手触りだった。
9	ザラつきが強かったから。所々、小さなつぶつぶがある？
10	ざらつきを感じた。
11	
12	しっとり感が少なかった。
13	あまり長い時間さわりたくはないと思いました。
14	ザラザラだけど滑らか。
15	滑らなそう。
16	ざらつきを感じました。
17	少しざらざら。しっとりしているなと感じた。
18	あらめのやすりを触っているみたいだった。かべみたい。
19	少しザラザラ。コンクリートを触っている感じ。温度はないが、少し湿った印象。滑りにくい。
20	くだものの表面のような湿り気を感じた。

J

ID	Comment
1	やすりみたいに、皮ふが削られていく感じがした。
2	サンプル表面がとてもでこぼこしているように感じた。
3	一番表面に粗さを感じた。冷たかった。摩擦が強いと思った。
4	表面がザラザラしてた。
5	ザラザラ。つっかかる。
6	粗さをすごく感じた。人工芝生とかホットカーペットのような硬めのカーペットみたいな感じがした。
7	アスファルトとか、そういう感じ。
8	すごくざらざらしていた。ふすま等に使われる布のイメージ。
9	粗いつぶを感じた。ザラザラしていた。
10	ざらざらとしていた。手に残る感じがした。
11	１番さらさらしていたように思う。
12	ざらざらしていたけど、くっつく感じがした。
13	目の粗いゴムをさわっている感じがした。
14	ぬるぬる。
15	何か削れそう。
16	つめやすりのような触感がしました。
17	ざらざらしていた。すべりはあまりよくないと感じた。
18	でこぼこしていなかったら触り心地がよかったと思う。
19	目が粗く、ザラザラで冷たい感じ。動かしても温かくはならない。手になじまないが少し水気？思いっきりこすったらすり傷になりそう。
20	かたい。ざらざらしてるけど痛くない。

K

ID	Comment
1	毛ばだっていて、フェルトをさわっているようだった。
2	ふわふわで指を動かしやすかった。
3	コットンの表面をなでているようだった。あたたかみを感じた。乾いていて、さわさわしていた。
4	サンプルがかたかったのと乾いていたために、スベスベ・滑らかな感じはしたものの湿っ気（しっとり感）は感じられなかった。
5	布みたい（じゅうたんみたいな手触り）。
6	毛が並んでいる！って感じがした。こする方向によってなめらかだったり、少し抵抗感を感じたりした。
7	ざらざらとさらさらの間で、最初だけふわっと。
8	フェルトとコットンのような手触りだった。少しざらざらしていた。
9	少しふわふわしていた。しっとり感はなかった。
10	指を置いた瞬間は1つ前よりもふわふわとした感じがした。毛に逆らうようになぞっているときはざらつきを感じた。
11	さわる方向による。粗さ。
12	右から左に移動する時に少しなめらかさを感じた。
13	さらさらしてた。
14	毛が短くて固く感じた。キウイみたいだった。
15	滑らかな毛。
16	フェルトを少しやわらかくしたような感じがしました。
17	やわらかくてざらざらした。
18	ボサボサしているように思った。
19	触った瞬間はフワッとした感じ。小動物の毛なみみたいな印象。右に動かすと逆毛をなでている感じで少し固さがあったが、左に動かす分にはなめらか。少し
20	ひっかかりがあるけど滑らか。

L

ID	Comment
1	ガサガサしていて、ひからびている感触だった。
2	表面が粗めの生地のようでした。
3	粗く、あたたかみのある素材を感じた。しっとりや、なめらかとは思わなかった。
4	表面が粗めの生地のようでした。パサパサ・ザラザラしていた。乾いた感じがした。
5	やわらかい毛、ふわふわしている。
6	触った瞬間はふわっという感じ。こすり始めてからはふさふさしてた。繊維感？毛を触っているなという感じ。
7	表面に細かい毛があって若干チクチクした。
8	3回目？くらいのサンプルと似ていた。コットンみたいな感じ。
9	固い紙を細かく破いて触ったときの感触。ガサガサ。
10	指を置いた瞬間はふわりと感じた。動かしている時は少しざらつきを感じた。
11	
12	右から左はなめらかに感じ、左から右に移動するときはすこし粗さを感じた。
13	左右に動かしている時、一方向はさらっとしてて、もう一方向はがりがり？ざらざら？してた。
14	毛は少しかたく感じた。温かみを感じた。
15	パサパサしていた。動物（ウリボー）の毛皮っぽい。
16	片方に指を滑らせる時に粗さを感じました。
17	ざらざらふわふわしていた。ぬれている感じはまったくしない。
18	ぬくもりを感じたような気がする。もふもふする素材が好きだった。
19	ザラザラ。毛が短いじゅうたんをなでているような感じ。くっつく感じはなく、乾いてサラサラしている。左に動かしたときはかたさは感じなかったが、左に動かし
20	ざらざらしていたけど、方向によってはなめらかだった。

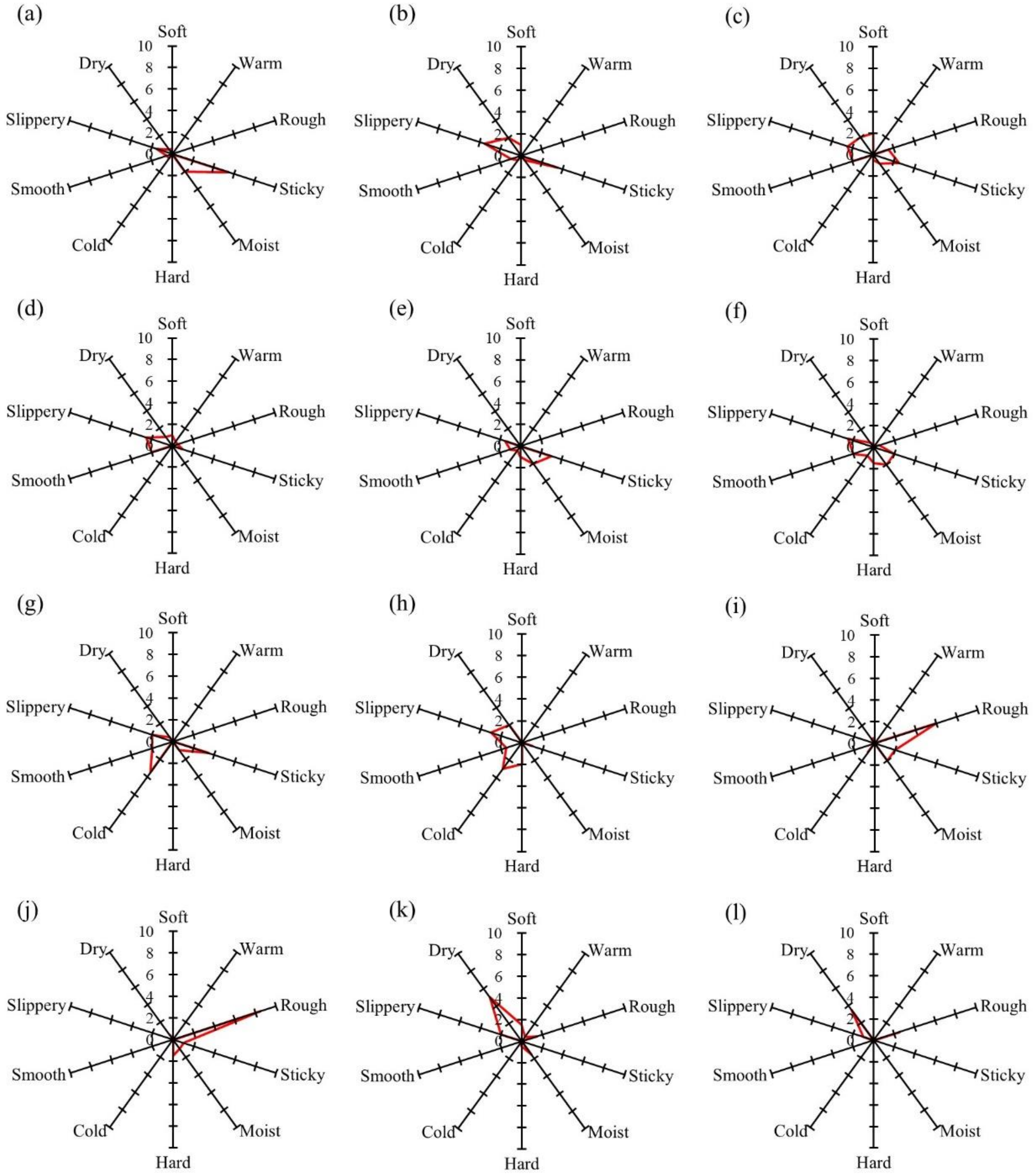

Figure S4 Appearance rate of the words on each tactile dimension in the subject's comment: (a) powder A (alkyl-silane-treated sericite), (b) B (*N′*-lauroyl-*L*-lysine), (c) artificial leather C (polyester/polyurethane), (d) D (polyester), (e) resin E (acrylic resin), (f) F (PTFE), (g) metals G (aluminum), (h) H (copper), (i) silicone rubbers I (silicone rubber on which surface structure of emery cloth #80 was transferred), (j) J (silicone rubber on which surface structure of emery cloth #40 was transferred), (k) cloth K (Japanese cashmere) and (l) L (Swiss cashmere).

Table S4 Physical properties.

Material	μ_s	μ_k	Delay time δ	$\mu_s - \mu_k$	Friction variation value	Surface roughness						Surface tension / mN m^{-1}	Young's modulus / MPa	Thermal conductivity / W mK^{-1}	Water content / %
						R_a / μm *	R_z / μm *	R_a / μm **	R_z / μm **	R_a / μm ***	R_z / μm ***				
A	0.73	0.55	0.02	0.18	0.009	11.0	127	5.81	67.9	5.46	29.9	100	0.26	0.62	0.31
B	0.55	0.39	0.02	0.15	0.011	15.0	157	6.64	54.4	5.51	36.4	42.0	0.21	0.24	0.13
C	0.45	0.41	0.02	0.05	0.007	44.7	509	34.6	261	29.6	226	42.0	0.39	0.03	0.58
D	0.50	0.56	0.02	-0.07	0.006	16.5	326	12.1	122	9.34	68.0	42.0	0.45	0.04	0.80
E	2.31	2.04	0.04	0.27	0.053	0.36	3.39	0.12	0.96	0.01	0.08	39.0	30.2×10^{2}	0.17	0.58
F	0.28	0.17	0.01	0.11	0.003	7.12	56.8	2.22	22.4	0.26	2.51	18.5	476	0.25	0.01
G	1.31	1.14	0.03	0.17	0.084	5.69	54.8	1.14	10.1	0.14	1.48	873.0	7.50×10^{4}	156	0.00
H	0.58	0.46	0.02	0.12	0.04	7.70	69.8	1.82	13.5	0.26	2.60	1300.0	1.30×10^{5}	275	0.00
I	0.72	0.74	0.02	-0.02	0.021	40.6	363	32.8	219	20.2	114	24.0	3.35	0.65	0.17
J	0.65	0.70	0.02	-0.10	0.021	116	741	104	451	78.2	344	24.0	3.35	0.65	0.09
K	0.47	0.42	0.02	0.05	0.008	105	955	86.9	670	72.9	457	35.55	0.33	0.04	7.38
L	0.55	0.46	0.02	0.09	0.01	93.9	721	79.2	525	59.1	446	35.55	0.18	0.03	6.91

The symbol *, **, *** mean the difference in the measurement magnification of the surface roughness: * , **, *** are 5, 10, 20 magnifications, respectively.
